# Designing a conceptual framework for misinformation on social media: a qualitative study on COVID-19

**DOI:** 10.1186/s13104-021-05822-2

**Published:** 2021-11-02

**Authors:** Peivand Bastani, Seyyed Mostafa Hakimzadeh, Mohammad Amin Bahrami

**Affiliations:** 1 Health Human Resources Research Center, School of Management and Medical Informatics, Shiraz University of Medical Sciences, Shiraz, Iran; 2 Department of Healthcare Management, School of Management and Medical Informatics, Shiraz University of Medical Sciences, Shiraz, Iran; 3 Baqiat Allah University of Medical Sciences, Tehran, Iran

**Keywords:** COVID-19, Coronavirus, Infectious disease, Outbreak, Misinformation, Infodemic

## Abstract

**Objective:**

This study was aimed to present a conceptual framework about the misinformation surrounding COVID-19 outbreak in Iran. For this purpose, discourse analysis of two of the most common social virtual networks were conducted via a four step approach as follows: defining the research question and selecting the content of analysis, gathering information and theory on the context, content analysis for establishing the themes and patterns and, presenting the results and drawing conclusions.

**Results:**

Cultural factors, demand pressure for information during the crisis, the easiness of information dissemination via social networks, marketing incentives and the poor legal supervision of online content are the main reasons for misinformation dissemination. Disease statistics; treatments and prevention are the main subjective categories of releasing misinformation. The consequences of misinformation dissemination include psychosocial, economic, health status, health system and ethical ones. The most recommended strategies for dealing with the issue could be divided into demand and supply-side strategies.

## Introduction

In December 2019, the novel coronavirus originated from Wuhan, China and quickly emerged as the greatest global public health threat [1–4]. Following this, scientists mobilized to investigate different aspects of the pandemic, including its potential consequences for societies using online data [5].

Social media as a quick available online data source in the midst of an outbreak was mentioned considering the speed of the outbreak [5, 6]. It has become an important medium for information dissemination during the pandemics and is playing a crucial role in health management [7]. During crisis, social media provides trusted sources for public, enables connectivity, advances remote learning, and even accelerates research [4, 8]. Chew and Eysenbach stated social media could be used for real-time infodemiology studies, providing a source of information for health authorities to respond to public concerns [9].

While social media play important roles in crisis management [6], it also comprises a disturbing role trough the widely dissemination of misinformation, and fake news which can make crisis management more difficult [7].

Misinformation is defined as information that is false or inaccurate and not supported by scientific evidence [10]. Current literature shows that during the outbreak of COVID-19 a large volume of inaccurate information is releasing through social media [11] that makes governments face the challenge of an “infodemic,” which causes people to experience difficulties in finding credible, evidence-based and trustworthy information [6, 11]. A recent study has reported that during the global pandemic, COVID-19 misinformation can greatly affect public awareness, knowledge and behaviors [12].

Besides, researchers also noted that the medical misinformation content pertaining to the COVID-19 pandemic is being proliferated at a frightening rate on social media [13]. Shaw et al. stated that there will be a tsunami of information on social media [14]. Brennen et al. said that misinformation pertaining to the global health crisis COVID-19 pandemic generates a severe risk to public health [15]. Other studies reveal that misinformation in the context of COVID-19 include inaccurate information regarding the virus and its transmission, conspiracy theories, methods of prevention and treatment [16].

With the wide use of social media around the world including Iran, it is expected that the citizens are widely exposed to COVID-19 misinformation, through either active seeking of information or passive receiving of them. In fact, a defining characteristic of this pandemic has been the spread of misinformation such as WHO famously called the crisis not just a pandemic, but also an “infodemic”. Why and how misinformation spreads and has an impact on behaviors, beliefs and health outcomes is a complex and multidimensional phenomenon. Therefore, this study aimed to investigate the phenomenon from the discussions of Iranian health scientists using online data to provide insights for the health authorities to deal with the production, dissemination and use of misinformation.

## Main text

### Study design and data collection

A social media monitoring was conducted using the largest social media platforms WhatsApp and Telegram in Iran with approximately 42.8% and 42.4% of users, respectively [17].

We used a qualitative design to analyze the discussions of social media users about the content related to COVID-19 transferred via Iranian medical faculty members` groups in Telegram and WhatsApp from Feb 20 to March 20, 2020. All of these groups were composed of faculty members affiliated in Iranian Medical Universities all over the country and more than 1000 members have joined them. The basic sciences and clinical faculty members with the ranks of assistant, associate and full professor have joined these groups with different specialties. Most of them have PhD or clinical specialty. These groups are unofficial and all the faculties are free to join. All the group members were able to share a piece of information in the audio, video, or textual format. Also, the space of discussion about the contents of the shared information was available. The researchers as the members of these groups, only read and record all the contents and the discussions about them in an electronic form. In the course of data collection, researchers didn’t participate in discussions to be able to analyze the discourses and to avoid any bias. The present proposal was developed merely for this study. As it is mentioned in the ethical approval section, the whole study is approved by Shiraz University of Medical Sciences with the (Grant No. 22283). All the related data is presented only in this manuscript.

### Analysis and categorization of collected data:

Discourse analysis was applied for the content analysis of the discussions. According to the aim of discourse analysis, the written or spoken language about the social context can be studied [18]. For this purpose, four steps were conducted as follows:

I. Defining the research question and selecting the content of analysis. The research question was defined as “how and why the misinformation about COVID-19 disseminates via social online platforms and what are the negative consequences and best coping strategies”. Then, the content was selected for analysis. In this regard, 5 WhatsApp and Telegram groups containing medical faculty members all over the country were chosen and all the related content about COVID-19 was selected.
II. Gathering information and theory on the context. A rapid search on the databases of PubMed, Medline and Google scholar was conducted because of their free access in Iran applying these keywords: “misinformation”, “disinformation”, “false information”, “wrong information”, “fake news” “cause”, “incentive”, “stimulus”, “reason”, “typology” and “coping strategies” with the combination by “COVID-19” or “coronavirus”. Also, the governmental health guidelines and framework were retrieved to achieve the best related framework to the research question. Finally, a framework consisting of causes and incentives for disseminating misinformation, the process, outcomes, and strategies were chosen for deductive data analysis based on the concept of information management flow [18].
III. Content analysis for establishing the themes and patterns. A deductive framework analysis approach was applied to analyze the contents. This approach provides clear steps to follow and produces highly structured outputs of summarized data. Framework analysis was recommended for multidisciplinary health researches with managing large data sets where obtaining a holistic, descriptive overview of the entire data set is desirable including 4 stages as follows: transcription, familiarization, coding and developing a working analytical framework [19, 20]. In this regard the whole data was reviewed and the related codes which were best suited with the elements of the selected framework were extracted deductively.
IV. Presenting the results and drawing conclusions. The results were reviewed and presented in a table and conclusions were drawn based on the pre-stated framework. All the analysis was conducted by the researchers that have no conflict of interest to the topic.

In order to assure the credibility of the analysis, a long-term involvement of the researchers was occurred during familiarization of the data and data analysis. Peer check and expert check were also applied to increase the rigor and trustworthiness of the analysis. The researchers’ reflexivity and lack of conflict of interest were also mentioned in data analysis.

## Results

Data analysis was reached to 4 main themes as follows: the incentives and reasons of generating misinformation; the process of producing misinformation; the negative consequences of spreading misinformation and the coping strategies (Table 1).

**Table 1:** Extracted themes and sub-themes regarding COVID-19 related misinformation on social media

Themes	Sub-themes
Incentives/reasons for misinformation producing	Cultural causes Demand pressure/need and demand for information in pandemic crisis The easiness of information dissemination via online networks Financial or marketing incentives Lack of legal supervision Lack of knowledge/ the weak an inadequate knowledge level of content producers Lack of knowledge in users/ lack of ability to assure the validity and credibility of the information Excitement and entertainment Corruption/ damage or destruction Wording errors Tendency to attract and increase the audience by rumor spread
Process of misinformation dissemination	Target audiences: All population groups
	Main channel: Social media
	Main subjective categories of misinformation: The statistics of the disease (incidence, prevalence, mortality rate, inpatient rate, recovery rate, predicting the future trends) The new treatments, vaccines and pharmaceuticals Preventive methods and personal and group protection tools Dietary recommendations and diets The ways of disease transformation Consumption of daily supplements Symptoms, clinical signs, prognosis, the cycle of the disease and Commune Period and the disease side effects New diagnosis methods Risk factors of the disease The nature of the Corona virus (e.g. The virus structure, its living context, the periodic and seasonal behavior of the virus) International and national documents and guidelines, governmental decisions for comforting and controlling the disease, fake instructions, public activities and resource allocation for disease control Fake religious or traditional narratives about the disease and its mechanism The health care system`s capabilities for confronting the disease Fake experiences of patients or healthcare providers Recommendation for consuming unconfirmed herbal treatments Designing and producing unstandardized medical equipment the same as masks, gloves, etc. at home Misinformation about the cities quarantine and traffic regulations
Negative consequences of misinformation dissemination	Psychosocial Health status Health system Economic Ethical
Coping strategies	Supply side strategies Demand side strategies

According to the present analysis, the members of monitored social networks believe that different incentives lead to the production and dissemination of COVID-19 related misinformation that could be categorized into 11 subthemes as presented in tale1. Regarding the process of misinformation dissemination, the analyzed discourses included three subthemes including the target audience of misinformation; the main online platforms used for disseminating the unsupported information and the types of main generated misinformation.

**Fig. 1:**
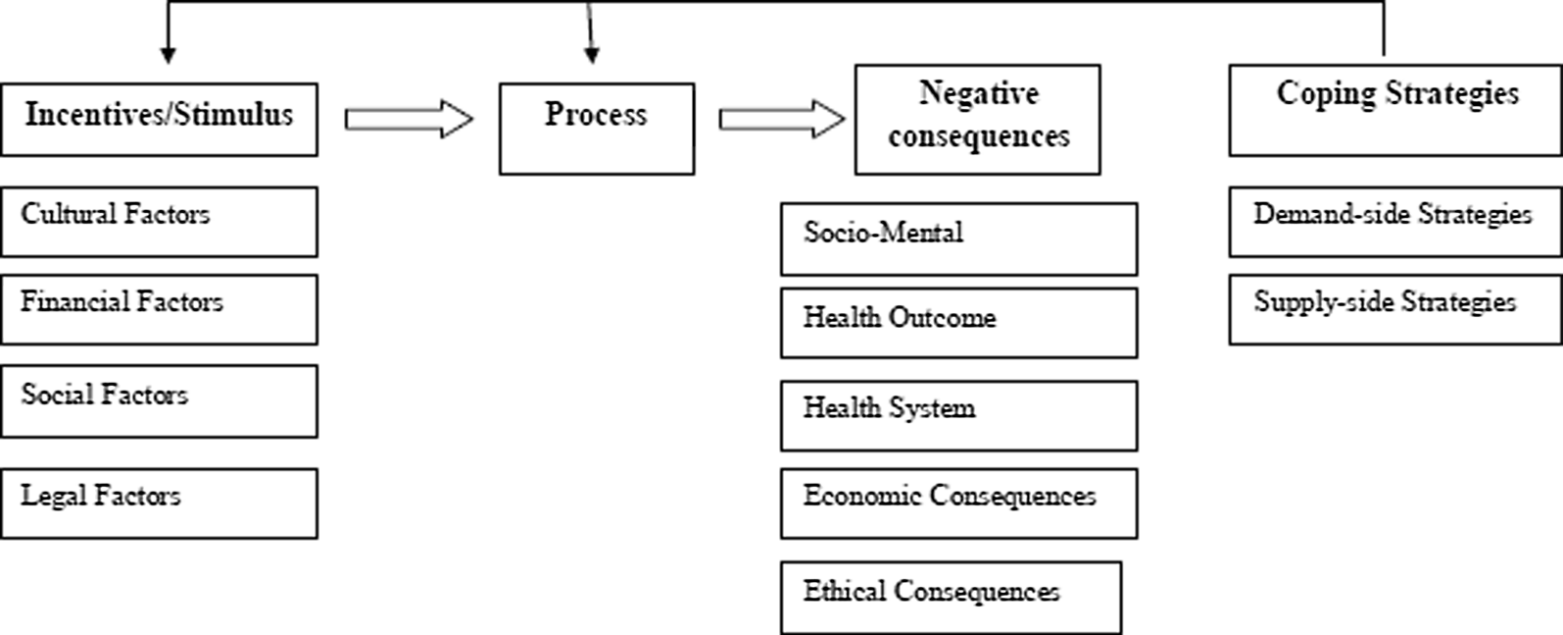
Thematic framework of study

Analysis also revealed that the broadly dissemination of misinformation regarding the epidemic imposes different negatives consequences on the country that can be categorized into psychosocial; health status; heath system; economic and ethical consequences. Finally, monitoring the target networks showed that a part of discourses is regarding the coping strategies to deal with the issues related to misinformation dissemination. Qualitative analysis revealed that the recommendations of members could be categorized into two groups of strategies named supply side strategies and demand side strategies that are presented in Table 1. Figure 1 shows the conceptual framework of misinformation proliferation during COVID-19 outbreak.

## Discussion

This study was aimed to qualitatively analyze the Iranian health experts' discourses about the COVID-19 related misinformation which are releasing on social media. The analyses were resulted to 4 main themes including:

### Incentives and reasons of generating misinformation

According to the results, the main reasons of generating misinformation about COVID-19 consist of cultural factors; pressure of demand during disease prevalence; easiness of dissemination of invalid information via online media; financial incentives and finally lack of proper supervision. Similar studies also show that during crisis, the public urgent need to health information and the over-demand of the information lead to generate great deal of information that most of it is false and invalid [21, 22]. Marketing and financial incentives are also considered as one of the motivators of disseminating misinformation in the other studies [22, 23].

### Process of generating misinformation

The present results show that during COVID-19 outbreak in Iran, the audiences of misinformation consist of all the population groups. Lee et al. have reported that overall, 67.78% of their respondents reported exposure to at least one COVID-19 misinformation item but misinformation exposure was associated with younger age, higher education levels and lower income [11]. As reported by Lee et al. [11] and Murphy [24] our findings showed that the social media was the main tools of transferring misinformation. The major topics/typology of the misinformation include: disease statistics, treatments, prevention and protection methods, dietary recommendations and methods of transferring the virus. Murphy has identified three common types of misinformation relating to COVID-19: false claims, conspiracy theories and pseudoscientific health therapies [24]. It seems that at the time of epidemic, the people want to know about the probability and severity of the illness very quickly and also they have a strong tendency to find the preventive and curative solutions [25].

### Consequences of generating misinformation

The consequences of disseminating misinformation about the disease from the experts` point of view include: the social-mental outcomes, inappropriate outcomes of health, outcomes related to the healthcare system and economical and ethical outcomes. Lee et al. have shown that misinformation exposure is associated with psychological distress including anxiety, depressive and posttraumatic stress disorder symptoms as well as misinformation belief [11]. Badell-Grau has found strong correlations between the volume of COVID-19–related search terms and per capita mortality and cases [26]. Ahinkora et al., stated that the dissemination of misinformation can powerfully impact people’s actions and change the value of the interventions employed by local governments through their health institutions and other stakeholders [27].

### Coping strategies

The coping strategies can be divided in two main demand-side and supply-side strategies. Barua et al., have recommended credibility evaluation of misinformation as the main recommendation for resilience of disastrous consequences of misinformation [28]. Ittefaq et al., suggested some recommendations to address misinformation regarding COVID-19 including putting more resources into the Perception Management Initiative (PMI); allocating funds and training media workers to fact-check information from online sources; promoting only authentic sources for information regarding COVID-19 [29]. Murphy, recommend three strategies including: train people how to identify and recognize fake news stories; stop tolerating pseudoscience health practices and Swamp the landscape with accurate information [24].

## Conclusion

This study helps health authorities in fighting with COVID-19 misinformation on social media providing insights about different aspects of the phenomenon including the stimulus; typology and coping strategies. Considering the potential of misinformation and fake news to undermine global efforts in the COVID-19 management, findings of this study provides a useful tool for health authorities to counter the proliferation of misinformation and overcome the infodemic.

## Limitations

This study has some limitations. First of all, the situation associated with the COVID-19 pandemic is rapidly evolving, so with the present knowledge this study tried to synthesize a framework for misinformation. Secondly, the study is run in the transferred contents of a definite society, and finally, only the contents of two social media are analyzed.

## Data Availability

The datasets used and/or analysed during the current study available from the corresponding author on reasonable request.

## Abbreviation

WHO: World Health Organization
